# Supplementary material for: Assessment of the Hematopoietic Differentiation Potential of Human Pluripotent Stem Cells in 2D and 3D Culture Systems
Source: Cells. 2021 Oct 23;10(11):2858. doi: 10.3390/cells10112858 (PMC8616232; doi:10.3390/cells10112858)
Supplement: Supplementary file 1 [file cells-10-02858-s001.zip › cells-1380870-supplementary.pdf]

Supplementary Files

Assessment of the Hematopoietic Differentiation potential of human pluripotent Stem Cells in 2D and 3D Culture Systems

Mora-Roldan German Atzin., Ramírez Dalia., Pelayo Rosana., Gazarian Karlen.

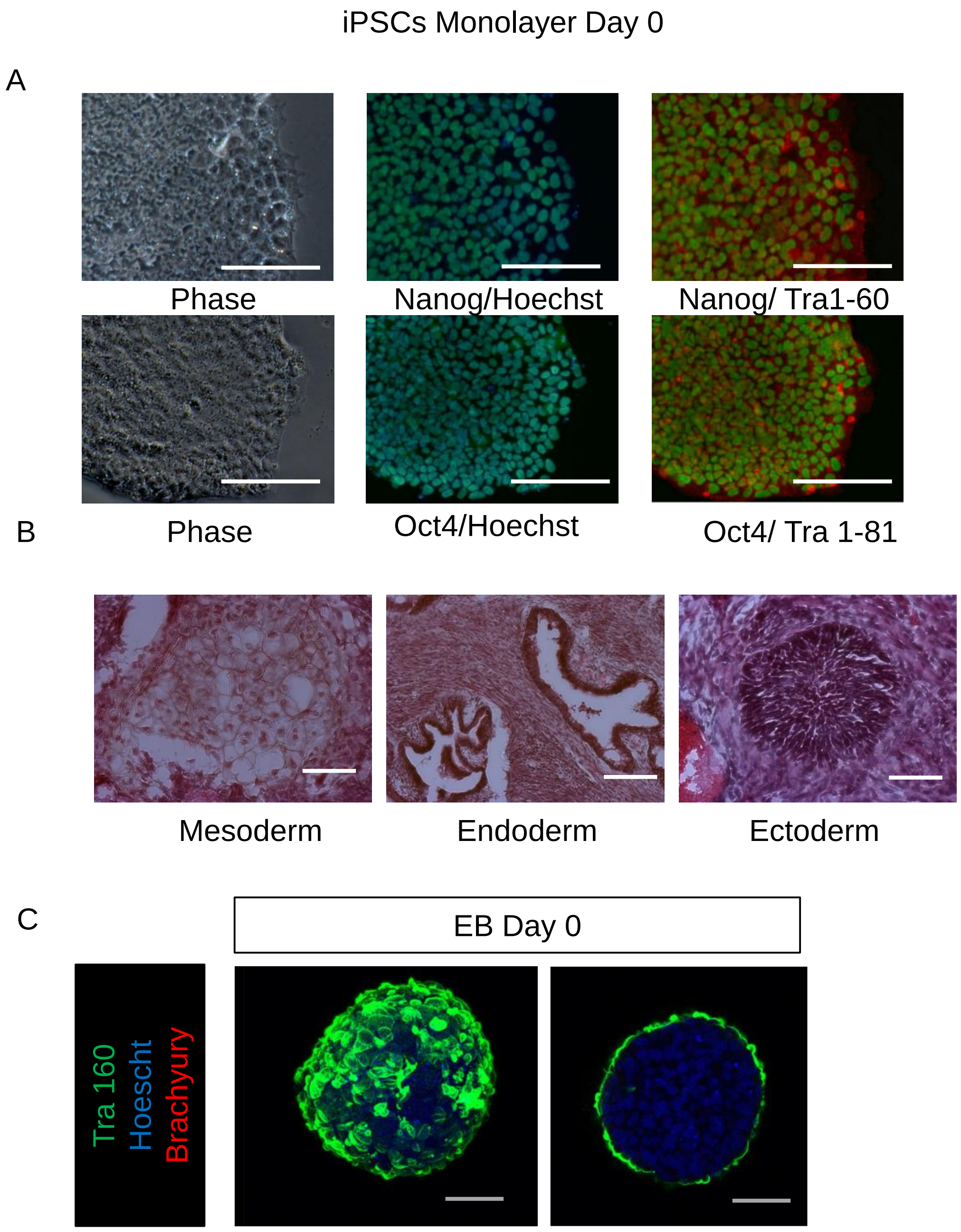

Fig. S1. Pluripotency assesment of iPSCs in Monolayer and EB. (A) Phase contrast photograhy and Co-expression of nanog, Oct4 and Tra1-60 pluripotency markers. (B)Teratoma formation in Nu/Nu mice; cells were introduced via subcutaneous hip injection and after 30 days tissue was dissected and stained, cells from the three germ layers were identifed. (C) Pluripotency marker Tra-160 of an iPSC colony in 3D, cells are negative for mesodermal marker T on Day 0.

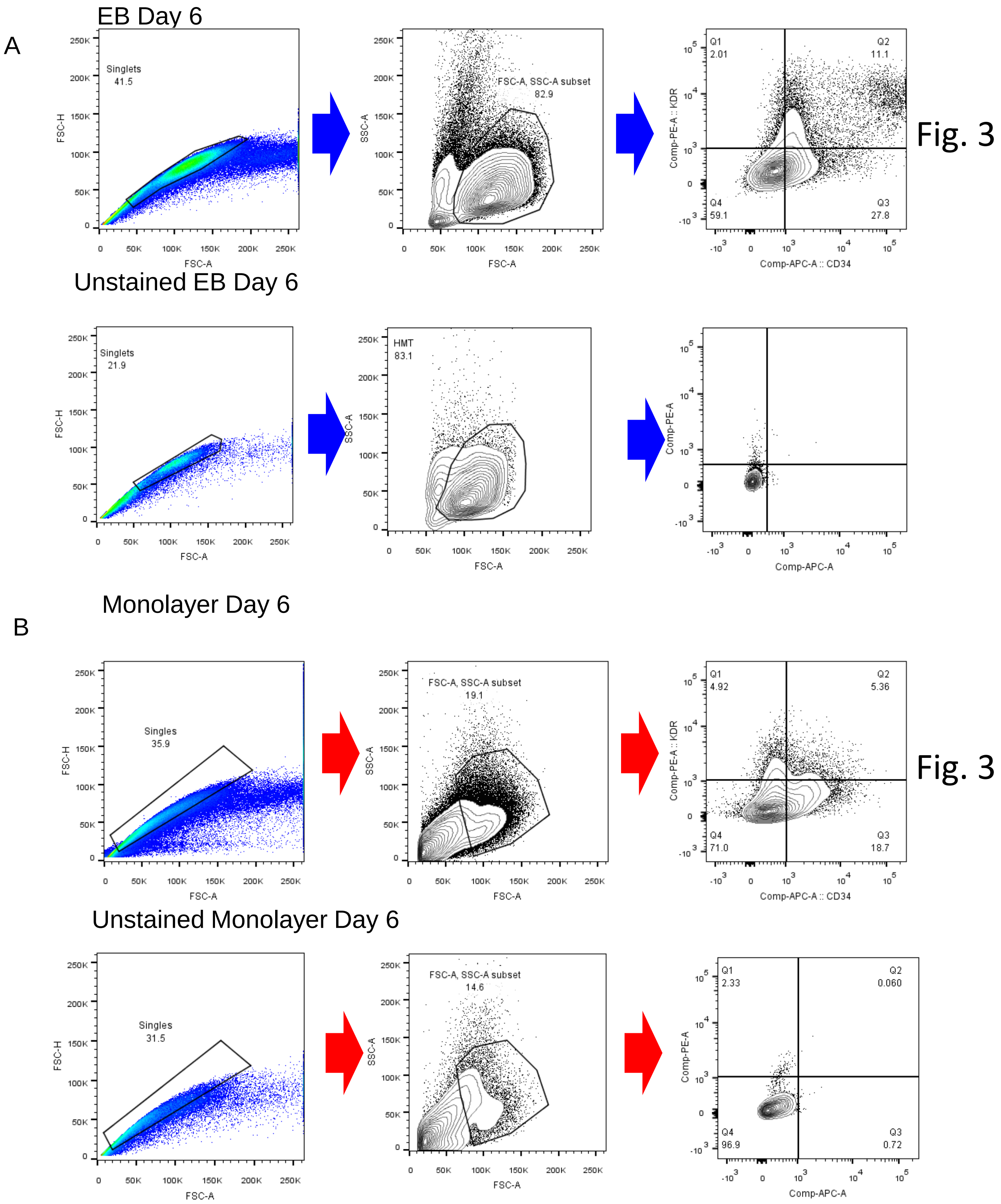

Fig. S2. Unstained controls and gating strategy for the comparison of EB/3D and 2D/Monolayer conditions. Unstained controls and gating strategy description for representetive images in figure 3.

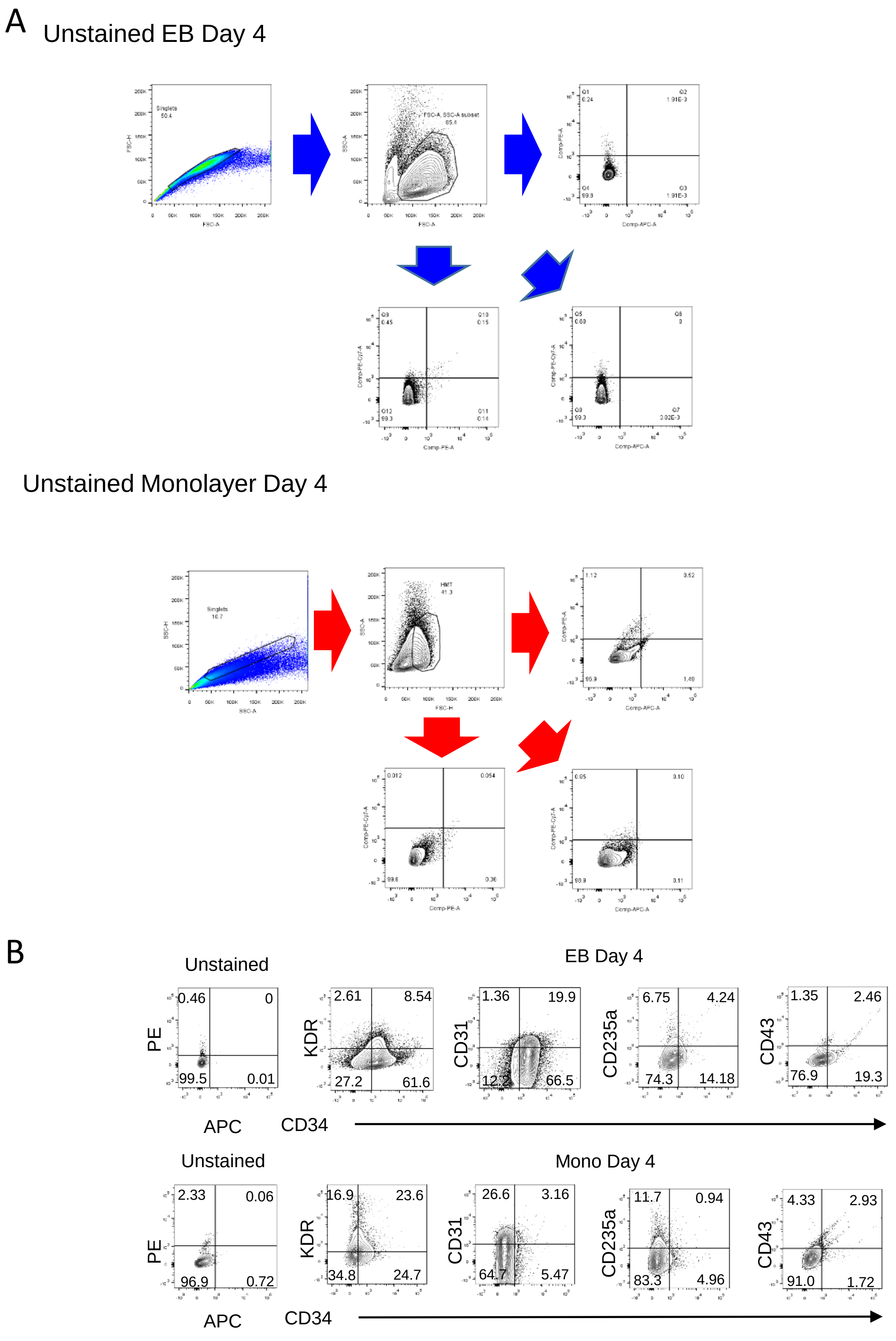

Fig. S3. Unstained controls for the comparison of EB/3D and 2D/Monolayer conditions. Unstained controls and gating strategy description for representative images in B.. (B) Representative data of Flow cytometry analysis of unstained control cells and cells co-expressing CD34 with the hematopoietic markers CD31 (Endothelium), CD235a (Primitive Hematopoiesis) and CD43 (hematopoietic progenitors) in 3D/EB and 2D/Monolayer culture formats.

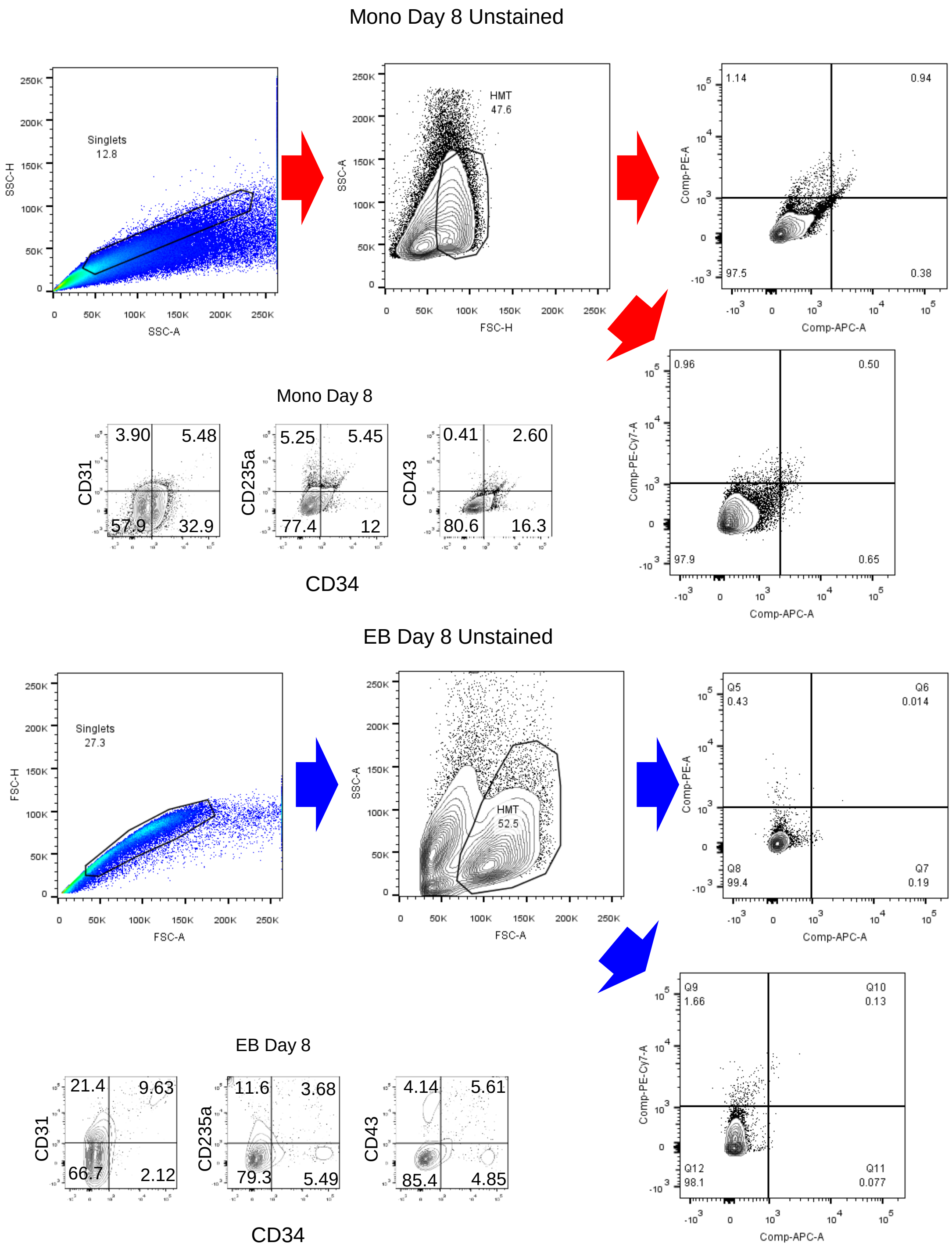

Fig. S4. Comparison of EB/3D and 2D/Monolayer conditions. Unstained controls and gating strategy description for Day 8 cells in both conditions.

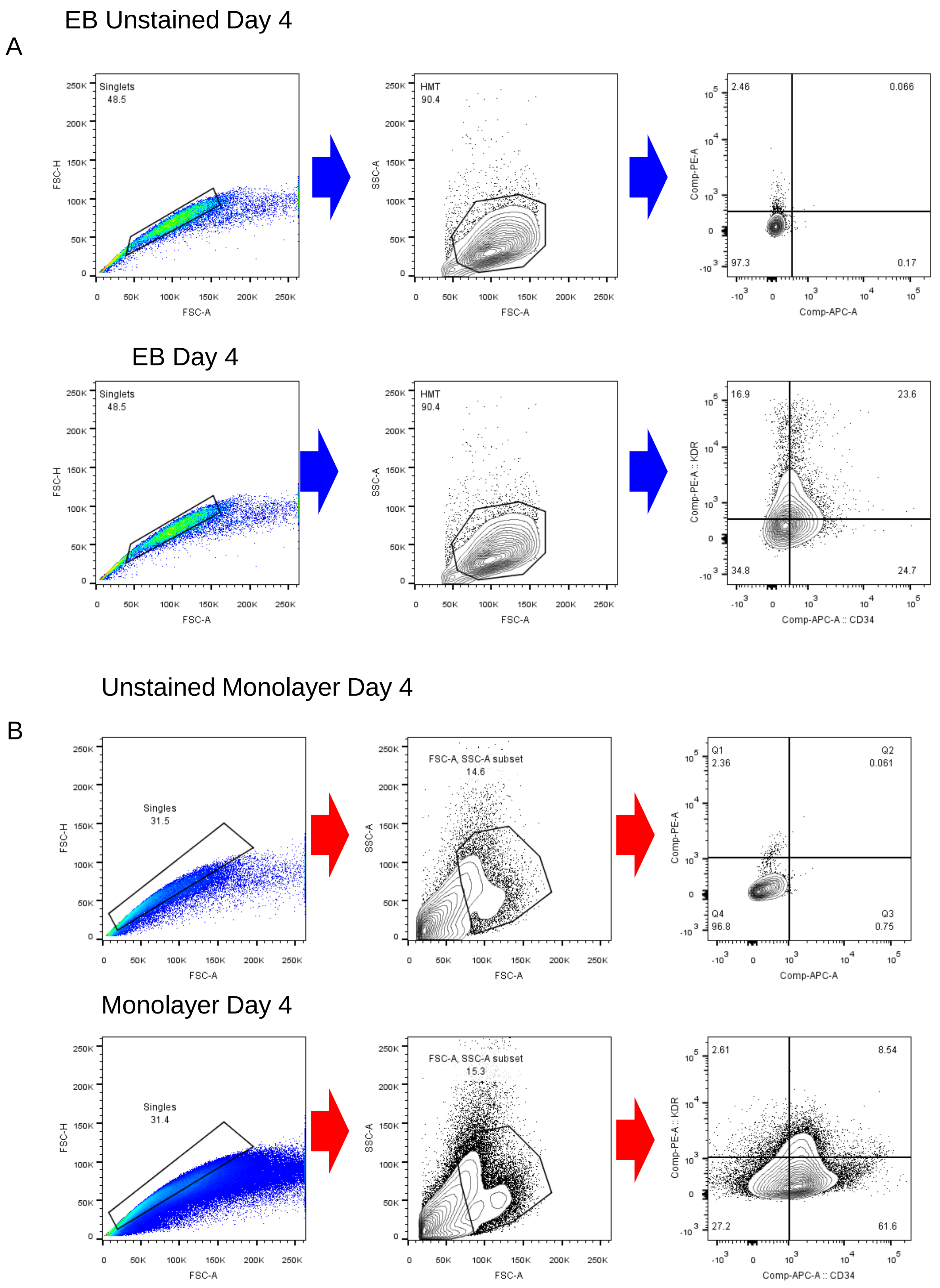

Fig. S5. Comparison of EB/3D and 2D/Monolayer conditions. Unstained controls and gating strategy description for Day 4 cells in both conditions.

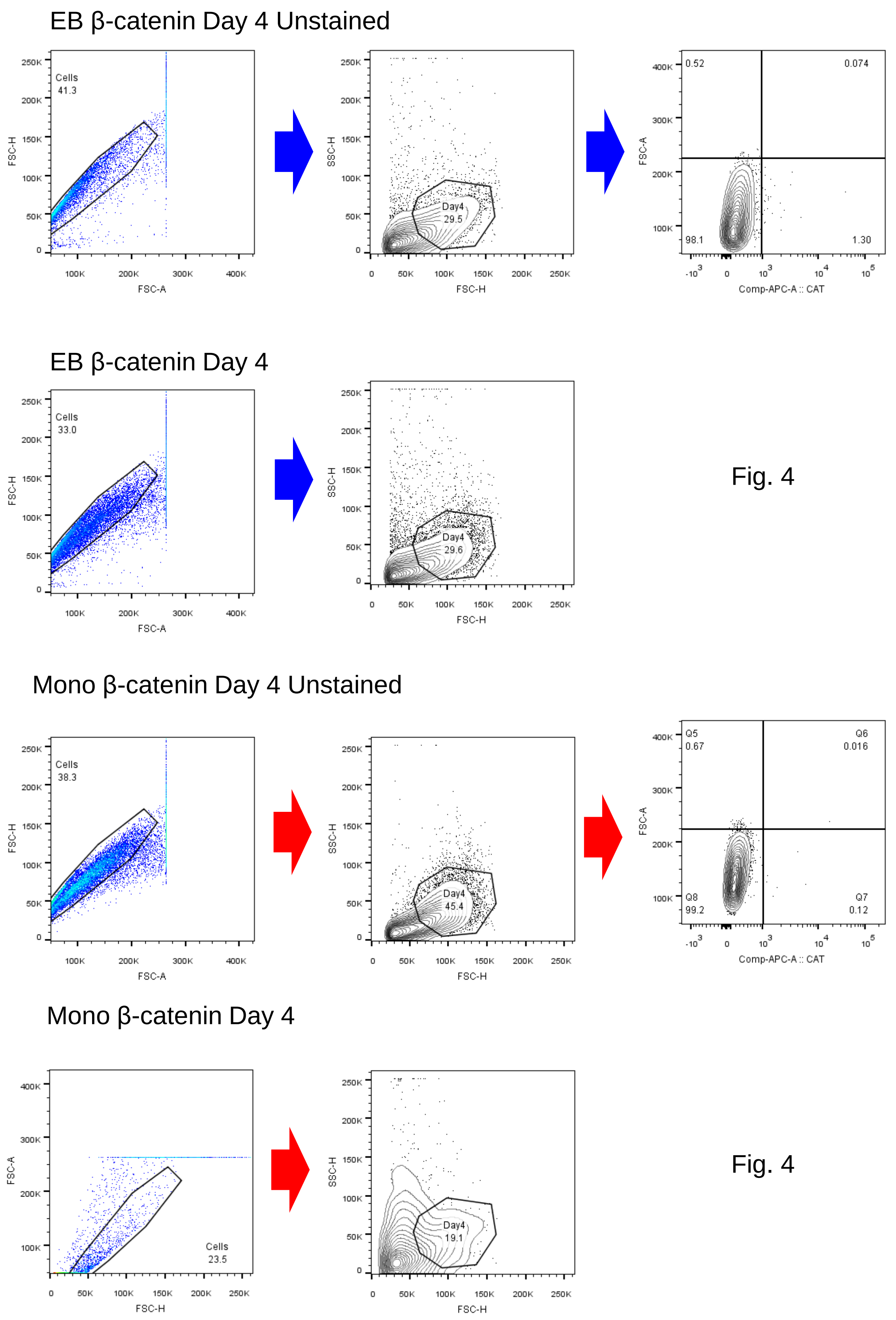

Fig. S6. Comparison of EB/3D and 2D/Monolayer conditions. Unstained controls and gating strategy description for Day 4 cells in both conditions, data of the Figure 4 in the main text.

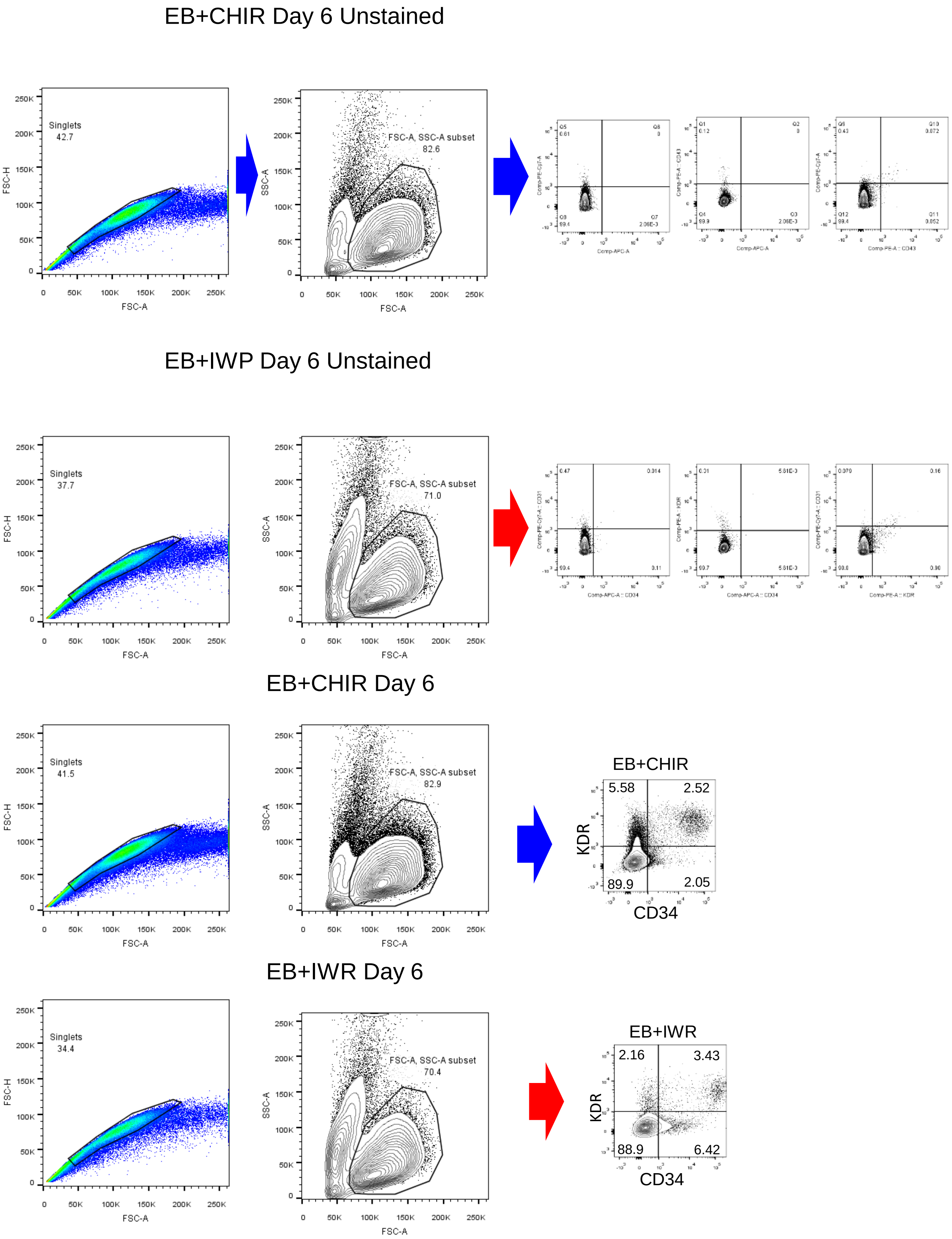

Fig. S7. Comparison of EB/3D and 2D/Monolayer conditions. Unstained controls and gating strategy description for Day 4 cells in both conditions. Data of the figure 4 in the main text.

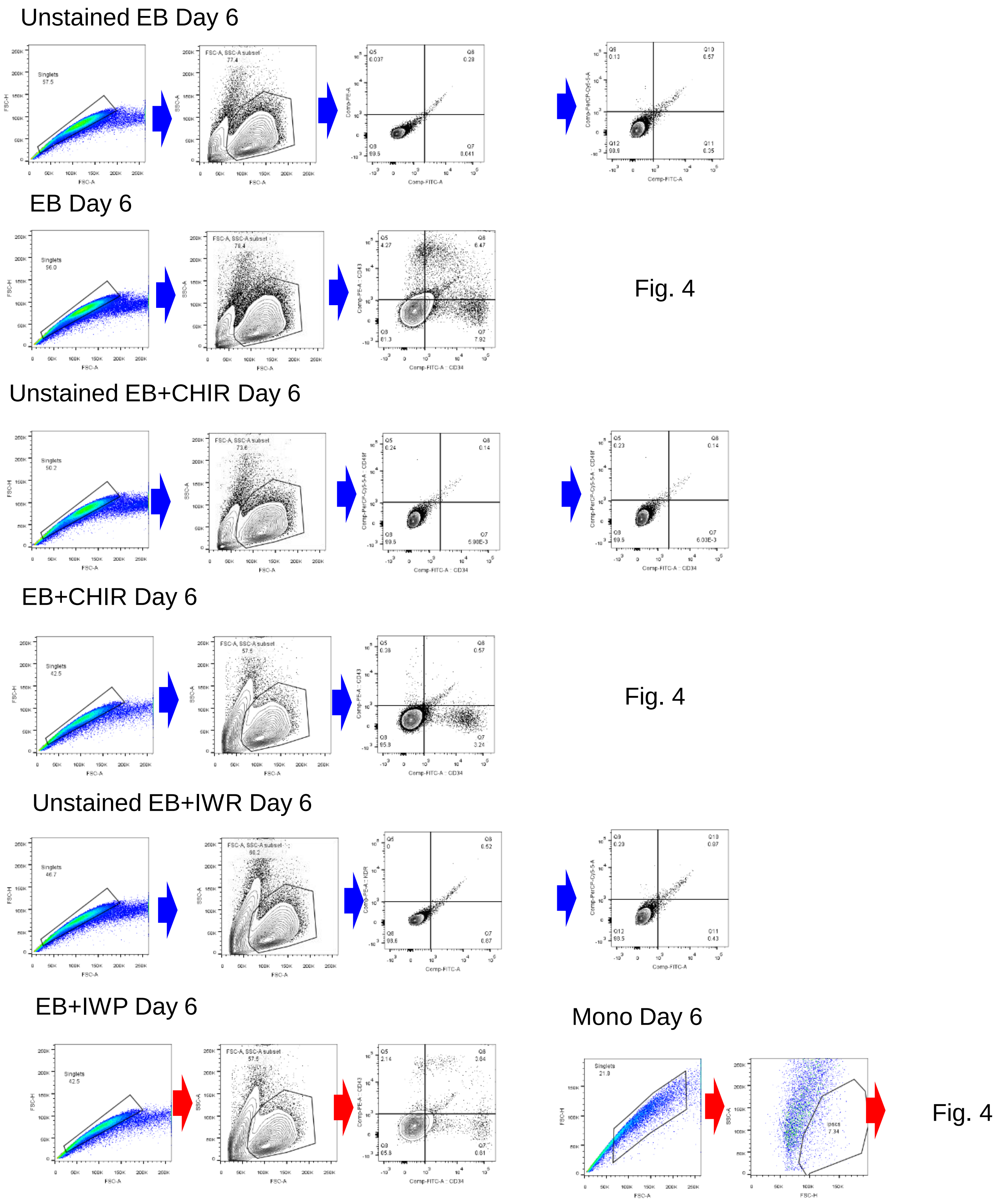

Fig. S8. Comparison of EB/3D and 2D/Monolayer conditions. Unstained controls and gating strategy description for Day 4 cells in both conditions. Data of figure 4 in the main text

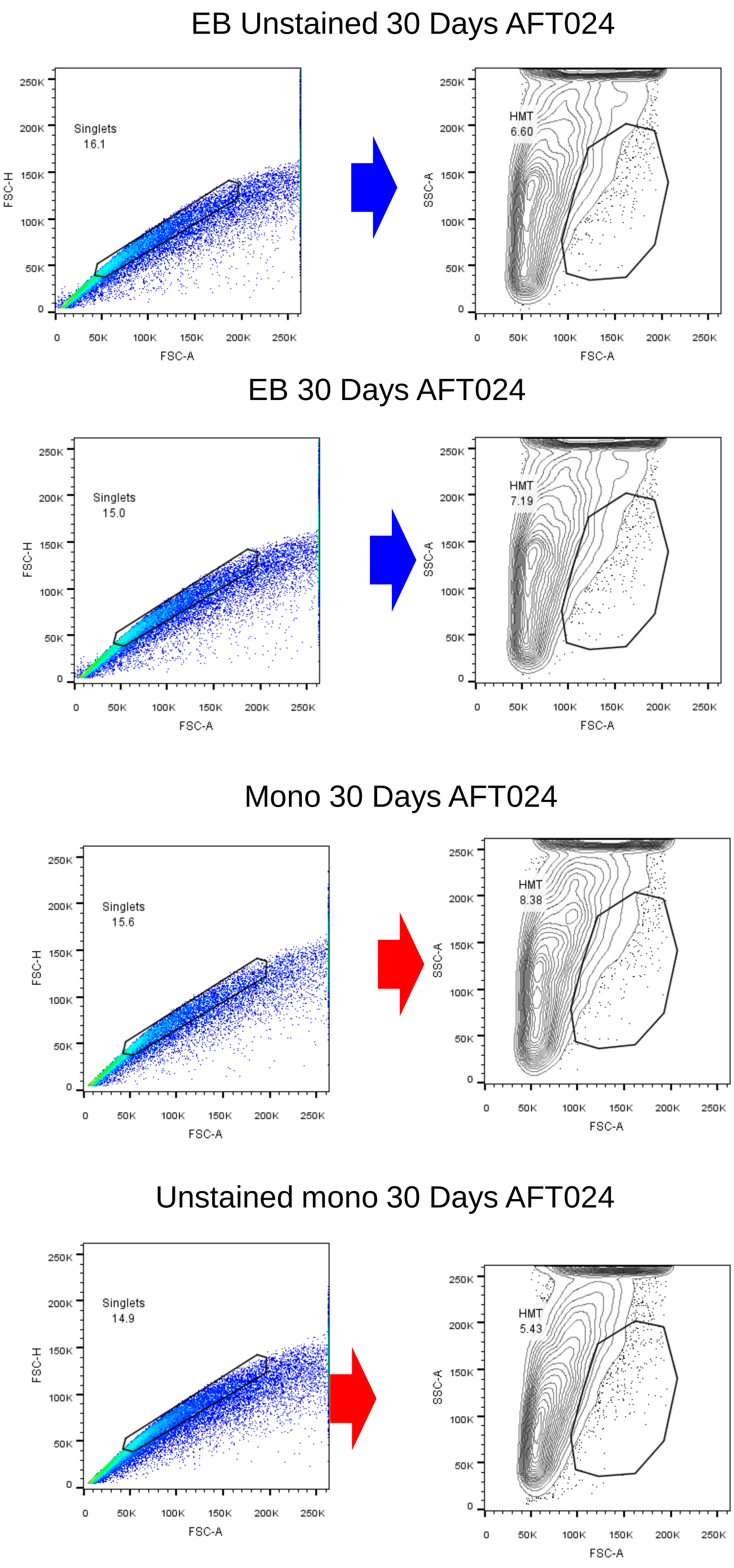

Fig. S9. Comparison of EB/3D and 2D/Monolayer conditions. Unstained controls and gating strategy description for Day 4 cells in both conditions.

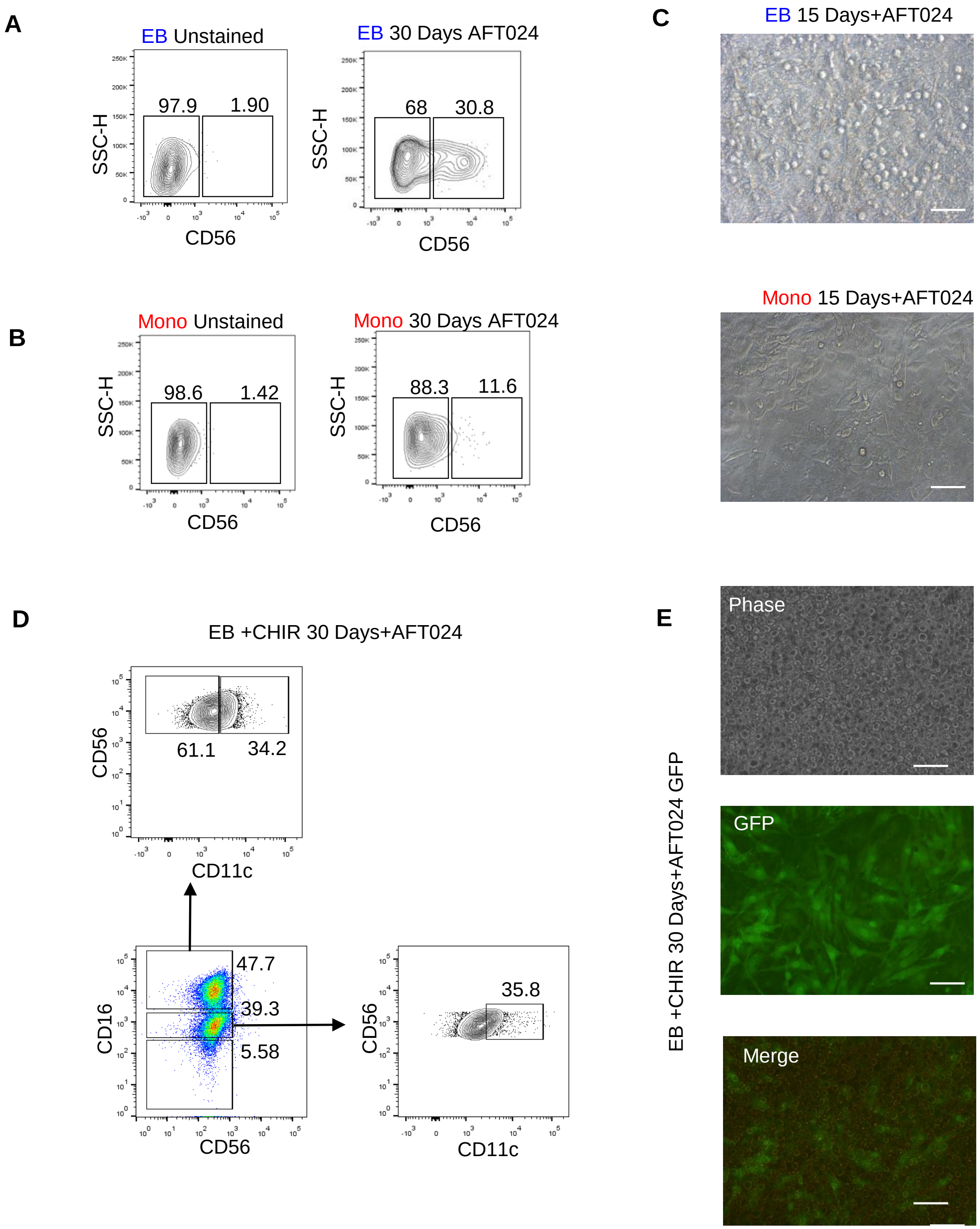

Figure S10. Day 6 cells after 30 days of differentiation on AFT024 cells. (A, B) CD56-positive cells and unstained controls, respectively. (C) Phase contrast images of the disaggregated day 6 cells cultured on AFT024gfp for 30 days in EB and Monolayer conditions. (D) CD56 and CD11c -positive day 6 3D/EB+CHIR stimulated cells. (E) Phase contrast images of disaggregated 3D/EB+CHIR day 6 cells cultured in AFT024gfp.

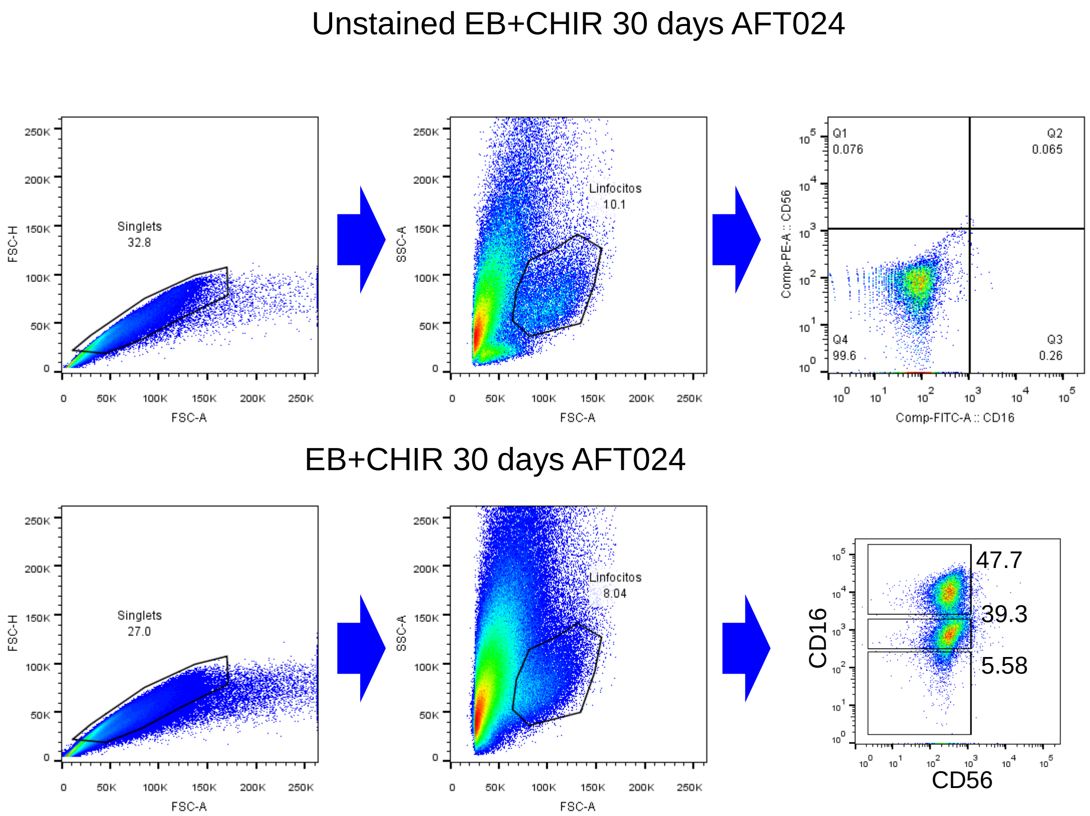

Fig S11. 3D condition supports NK-like Phenotype when stimualted with CHIR99021

Supplementary Table S1. Primers used for evaluation of mesodermal markers by qPCR, showing gene name, accesión number and secuences used.

Gene	Accesion number	Primers
KDR f<br>KDR r	NM_002244	GGCCCAATAATCAGAGTGGCA<br>CCAGTGTCATTTCCGATCACTTT
PDGFRa f<br>PDGFRa r	NM_006206	TGGCAGTACCCCATGTCTGAA<br>CCAAGACCGTCACAAAAAGGC
Brachyury f<br>Brachyury r	NM_003181	TATGAGCCTCGAATCCACATAGT<br>CCTCGTTCTGATAAGCAGTCAC
Sox17 f<br>Sox17 r	NM_022454	GTGGACCGCACGGAATTTG<br>GGAGATTCACACCGGAGTCA
BMP4 f<br>BMP4 r	NM_001202	ATGATTCCTGGTAACCGAATGC<br>CCCCGTCTCAGGTATCAAACT
Axin2 f<br>Axin2 r	NM_004655	CAACACCAGGCGGAACGAA<br>GCCCAATAAGGAGTGTAAGGACT

Supplementary Table S2. List of antibodies used for flow cytometry and immunoflorescence

Antigen	Brand/Catalog
Oct3/4	Cellsignaling#2750
Nanog	Cellsignaling# 4903S
Tra1-60 PE	Stemgent Code: 09-0010
CD 34 APC	Biolegend 343608
CD31 PE/Cy7	Biolegend 102418
CD43 PE	Miltenyi Biotech 130-100-094
CD235a FITC	Miltenyi Biotech 130-117-800
CD309 (KDR)-PE	Miltenyi Biotech 130-120-620
Brachyury-FITC	Millipore FCMAB302F
β-Catenin-<br>AF-647	Biolegend 844601<br>Abcam ab150115
Anti-Rabbit IgG Alexa488	Invitrogen Catalog # A27034
Anti-Rabbit IgG Alexa594	Invitrogen Cat # A32740
Anti-Mouse IgG+IgM Alexa488	Invitrogen Cat # A32723
